# Adapting an Evidence-based Physical Activity Program for The REJOIN Trial for Older Breast Cancer Survivors

**DOI:** 10.1093/geroni/igab046.2879

**Published:** 2021-12-17

**Authors:** Shirley Bluethmann, Eileen Flores, Meghan Grotte, Jared Heitezenrater, Cristina Truica, Kathryn Schmitz

**Affiliations:** 1 Penn State College of Medicine, Hershey, Pennsylvania, United States; 2 Penn State College of Medecine, Penn State College of Medicine/ Hershey, Pennsylvania, United States; 3 Penn State College of Medicine, Penn State Cancer Institute, Pennsylvania, United States

## Abstract

Purpose: Physical activity (PA) is a recommended part of breast cancer survivorship. PA promotes survival and mitigates symptoms in older breast cancer survivors (BCS), especially in reducing joint pain associated with adjuvant hormonal treatment. The purpose of this report is to describe adaptations to Fit & Strong!, an evidence-based curriculum, to meet the needs of older BCS. Methods: First, we reviewed all educational materials with scientific experts, including specialists in breast and exercise oncology. Next, we conducted semi-structured phone interviews with 3 BCS for an in-depth review of educational materials for the trial. All interviews were recorded and transcribed. Constant comparative analysis was used to identify themes and specify required technical changes. Subsequently, we recruited 3 new BCS to pre-test adapted materials and exercise sessions, complete a follow-up interview to refine our final product and rate acceptability with older BCS. Results: Overall, BCS found the materials and experience very acceptable (mean score of 9.5/10). Content changes included simplifying exercise instructions, prioritizing trial-specific content and updating photographs to be more age-appropriate. Due to COVID, the pre-test activity was conducted by Zoom and participants were given additional time and coaching to participate using this technology. BCS said they would prefer to exercise in person but reported the remote experience as very satisfactory. Conclusion: Our multi-step adaptation process provided an acceptable intervention to meet the needs of older BCS. Lessons learned will be applied to the forthcoming clinical trial, which will also be conducted remotely to maximize safety and access.

